# Prevention of feline leishmaniosis with an imidacloprid 10%/flumethrin 4.5% polymer matrix collar

**DOI:** 10.1186/s13071-017-2258-6

**Published:** 2017-07-14

**Authors:** Emanuele Brianti, Luigi Falsone, Ettore Napoli, Gabriella Gaglio, Salvatore Giannetto, Maria Grazia Pennisi, Vito Priolo, Maria Stefania Latrofa, Viviana Domenica Tarallo, Fabrizio Solari Basano, Roberto Nazzari, Katrin Deuster, Matthias Pollmeier, Laura Gulotta, Vito Colella, Filipe Dantas-Torres, Gioia Capelli, Domenico Otranto

**Affiliations:** 1 Dipartimento di Scienze Veterinarie, Università degli Studi di Messina, Messina, Italy; 2 Dipartimento di Medicina Veterinaria, Università degli Studi di Bari, Bari, Italy; 3 Arcoblu s.r.l., Milan, Italy; 4 Bayer Animal Health GmbH, Leverkusen, Germany; 5 Veterinary practitioner, Ambulatorio Veterinario S. Lucia, Via F. Crispi 56, Lipari, Italy; 6 Instituto Aggeu Magalhães, Fundação Oswaldo Cruz (Fiocruz), Recife, Brazil; 7 Instituto Zooprofilattico Sperimentale delle Venezie, Laboratorio di Parassitologia, Legnaro, Italy

**Keywords:** *Leishmania infantum*, Cat, Feline leishmaniosis, Prevention, Pyrethroids, Flumethrin

## Abstract

**Background:**

Leishmaniosis caused by *Leishmania infantum* is one of the most important vector-borne diseases affecting animals and humans worldwide. Dogs are considered main reservoirs of the zoonotic forms, though in the last years the role of cats as reservoirs has been increasingly investigated. Feline leishmaniosis (FeL) occurs in endemic areas and no specific preventive measures have been investigated so far. In this study the efficacy of a 10% imidacloprid/4.5% flumethrin polymer matrix collar, licensed for tick and flea prevention, has been assessed against FeL in a longitudinal study on 204 privately owned cats from the Aeolian islands (Sicily), an area highly endemic for the disease. From March to May 2015 [Study Day 0 (SD 0)], cats negative for FeL were collared (G1, *n* = 104) or left untreated (G2, *n* = 100). Diagnosis consisted of serology and qPCR on blood and conjunctival swabs, which were collected at baseline (SD 0) and at the end of the study (SD 360). Interim clinical examinations were performed on SD 210 (when collars were replaced in G1) and SD 270.

**Results:**

Of the 159 cats which completed the study, 5 in G1 and 20 in G2 were positive for *L. infantum* infection, in at least one of the diagnostic tests leading to a yearly crude incidence of 6.3% and 25.0% in G1 and G2, respectively (*P* = 0.0026). This translates into an efficacy of the collar of 75.0% in preventing feline *Leishmania* infection. The collar was generally well tolerated with no systemic adverse reactions and few local skin reactions were observed in the application area in four out of 104 treated cats (3.8%).

**Conclusions:**

The 10% imidacloprid/4.5% flumethrin collar significantly reduced the risk of *L. infantum* infection in cats. To our knowledge, this is the first study in which a preventative strategy against feline *Leishmania* infection is assessed under natural conditions. These findings close a gap in veterinary medicine, in that they confirm this collar as a tool in reducing the risk of *Leishmania* infection in cats. Such a preventative tool could contribute to the reduction of the risk of the disease in animal and in human populations when included in integrated leishmaniosis control programmes.

## Background

Leishmaniosis caused by *Leishmania infantum* (Kinetoplastida: Trypanosomatidae) is a vector-borne parasitic disease affecting animals and humans worldwide. The disease in humans is included amongst the most important neglected tropical diseases, with up to 0.4 and 1.2 million cases per year for visceral and cutaneous forms, respectively [1], and it has been the only tropical vector-borne disease endemic to southern Europe for decades [2]. Although dogs are regarded as primary reservoirs of *L. infantum* in many endemic areas, other domestic and wild animal species have been implicated in the epidemiology of the infection as secondary reservoirs [3, 4]. Since the first report of feline leishmaniosis (FeL) [5], the cat has been regarded as a resistant species and its involvement considered negligible to the epidemiology of the infection [6]. The main reason for this assumption was the low number of clinical cases in cats, especially when compared to that of dogs living in the same endemic areas [7–12]. In the last years, the development of feline medicine coupled with the employment of more refined serological and molecular protocols to diagnose the infection in cats have provided clues for a better understanding of FeL [13, 14]. Therefore, cases of FeL have been increasingly reported in areas endemic for canine leishmaniosis with prevalence rates up to 68.5% according to the cat population studied and diagnostic methodologies [14]. Also, in spite of the number of clinical cases that has always been considered marginal, reports of clinical conditions due to FeL are increasing either in cats suffering for immunodebilitating, concurrent infections such as feline immunodeficiency virus (FIV) and feline leukemia virus (FeLV), neoplastic diseases or in animals without any evidence of co-infections [14]. Remarkably, signs of FeL partially overlap those observed in diseased dogs with skin lesions and lymph node enlargement the most frequently reported [14–16]. Phlebotomine sand flies, the natural vectors of *L. infantum*, are generalist feeders and may take their blood meals from a variety of wild and domestic animals, including cats [17]. The infectiousness of *L. infantum-*infected cats has been demonstrated in xenodiagnosis studies for *Phlebotomus perniciosus* [18] and *Lutzomyia longipalpis* [19], two competent vectors. These data have ultimately provided further evidence on the possible role of cats as reservoirs for *L. infantum*. A recent study on vector-borne diseases (VBDs) of cats and dogs of the Aeolian Islands (Sicily, southern Italy), an endemic area for *L. infantum*, reported prevalence of 26% and 42% in cats and dogs, respectively, by serological and molecular methods [20]. In addition, up to 15% yearly incidence of *L. infantum* infection was assessed in cats exposed to one transmission season, indicating that, like dogs, cats living in endemic areas are exposed to the infection [20]. Cats are now recognized as a potential domestic reservoir of *L. infantum* and strategies to prevent infection in this animal species have been advocated [14, 16].

Currently, the most promising strategy for the prevention of *Leishmania* infection in dogs is the use of synthetic pyrethroids in different formulations (e.g. spot-on, collar and spray) with repellent properties against sand flies [4]. However, most of the pyrethroids, except for flumethrin, are toxic to cats [21] hampering studies on the prevention of *Leishmania* infection in this animal species [14, 16]. A polymer matrix collar containing a combination of 10% imidacloprid and 4.5% flumethrin (Seresto® collar, Bayer Animal Health GmbH, Monheim, Germany), thereafter referred to as the collar, has been recently registered for the use in cats for the prevention of flea and tick infestations associated with a repellent (anti-feeding) activity [22]. The same collar is also available for the control, up to 8 months, of ticks and fleas in dogs [23]; though not registered with a claim against sand flies, the collar proved to be highly effective (i.e. efficacy from 88.3 to 100%) in reducing the risk of *L. infantum* infection in dogs living in endemic areas [24–26].

In the present study, we investigated the efficacy of the collar in the prevention of feline *Leishmania* infection in a cohort of privately owned cats living in the Aeolian archipelago where FeL by *L. infantum* is highly endemic.

## Methods

### Study site and animals

The study was conducted from March 2015 to April 2016 in Lipari and Vulcano, two of the main islands of the Aeolian archipelago (Tyrrhenian Sea, Sicily, Italy, 38.4724°N, 14.9541°E), a geographical area recognized endemic for canine and feline VBDs and where an overall prevalence of 26% and an incidence of 15% of *L. infantum* infection were recorded in cats [20]. Animals were enrolled in the study from March to May 2015, before the beginning of the sand fly season and did not leave the study area or travel to other places. Cats enrolled in the study were 10 weeks or older, in satisfactory general health conditions, with a constant access to or living outdoors and negative for *L. infantum* infection by serology, quantitative real-time PCR (qPCR) and cytology (see below).

### Study design

This study was a Good Clinical Practice (VICH GL9 GCP) (http://www.vichsec.org) negatively controlled, partly blinded and randomised field study conducted on privately owned cats. The study protocol was approved by the Italian Ministry of Health and animals were included only after the signature of an informed consent by the owner. At the inclusion [Study Day 0 (SD 0)] cats were identified, physically examined, weighed and allocated to treatment groups (G1 = Seresto® collar for cats or G2 = untreated control) following a “per household” random allocation plan in order to avoid contacts between cats wearing the collar and untreated ones. Animals were sampled for blood and conjunctival swabs and those assigned to the G1 were treated with the collar according to the package leaflet. Briefly, the collar was fastened around the cat’s neck and adjusted according to label instructions until a comfortable fit was achieved, in that it was possible to insert two fingers between collar and neck when fastened. Animals assigned to the G2 group were left untreated and served as negative controls.

**Fig. 1:**
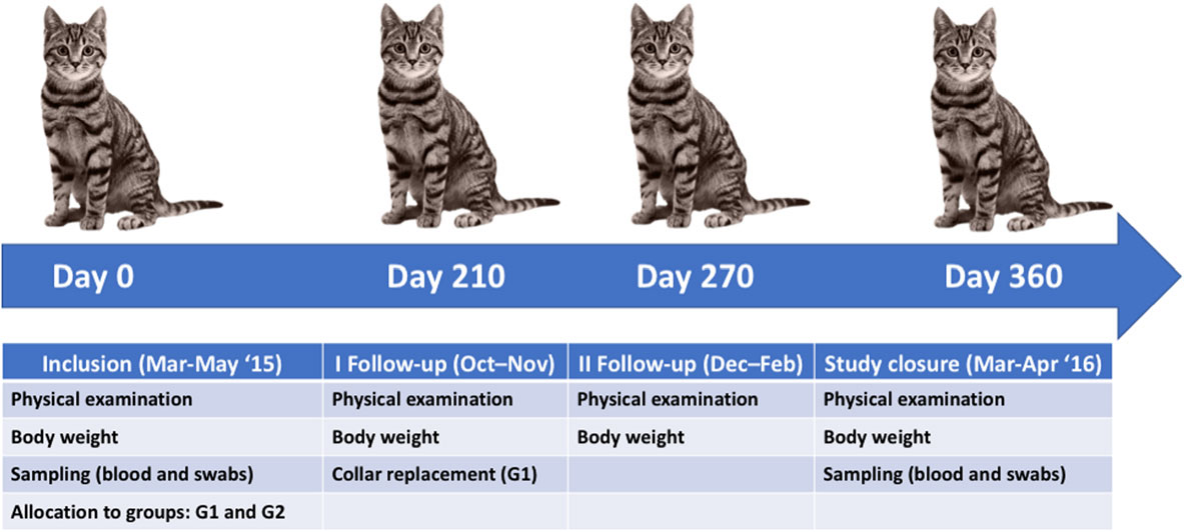
Time points of the study and scheduled activities

All the included cats were clinically examined and weighed at SDs 210, 270 and 360 (Fig. 1). In addition, at SD 360 (study closure) cats were sampled again for blood and conjunctival swabs. Collars in cats of the G1 group were replaced at SD 210 and at any time of the study in case of collar loss or damage. During the study cats remained with their owners and were managed as per normal routine without any containment measure or restriction. The owners were asked to observe their animals daily and to report, as soon as noticed, any abnormality in the general health of the animals as well as losses of or damages to the collar in cats of the G1 group. Any treatment with products with known efficacy against *L. infantum* vectors or ectoparasites was not allowed throughout the study. For animals in the G2 group, in the case of severe flea infestation, rescue treatment with Advantage® for cats (imidacloprid, Bayer Animal Health GmbH, Monheim, Germany) was allowed for animal welfare reasons.

### Sample collection and laboratory procedures

Blood samples of about 5 ml were collected from the jugular vein of which 2 ml were split into two anticoagulant tubes (K_3_ EDTA). From the first tube, two capillary tubes were filled up and centrifuged for buffy coat extraction and preparation of smears on glass slides. The remaining blood was processed for complete blood count using an automated blood cells counter (ProCyte Dx®, IDEXX Laboratories, Westbrook, Maine, USA). The blood in the second EDTA-tube was processed and analysed for the molecular diagnosis of *L. infantum*. Three millilitres of blood were stored in a tube with clot activator from which serum was obtained by centrifugation (1800× *g* for 10 min) and stored frozen (-20 °C) until analysis. Conjunctival swabs were collected for the diagnosis of *L. infantum* infection, using sterile cotton swabs manufactured for bacteriological isolation. One sample per eye was collected by rubbing the swab against the surface of the lower eyelid to collect the exfoliating cells. Conjunctival swabs were kept in sterile tubes and stored frozen (-20 °C) until analysis. Serum samples collected on SD 0 and SD 360 were tested for anti-*L. infantum* antibodies by using an immunofluorescence antibody test (IFAT) protocol, as described elsewhere [27]. The IFAT assay was prepared using conjugates specific for cats (anti-cat IgG; Sigma-Aldrich, St. Louis, Missouri, USA) and a positive control, obtained from the serum of a *L. infantum* diseased cat was included in each slide. Samples were scored as positive when they produced a clear cytoplasmatic and membrane fluorescence of promastigotes at a cut-off dilution of 1:80 for those on SD 360 (study closure), although animals with 1:40 dilution of sera collected on SD 0 (inclusion) were excluded from the study. Positive sera were titrated by serial dilutions until negative results. Blood and conjunctival swab samples collected on SD 0 and SD 360 were molecularly analysed for *L. infantum* by qPCR. Briefly, genomic DNA was extracted from blood and conjunctival swabs using the QIAamp DNA Micro Kit (Qiagen, Milan, Italy), following the producer’s recommendations. Thereafter, a fragment (120 bp) of the *L. infantum* minicircle kinetoplast DNA (kDNA) was amplified by qPCR using a protocol described elsewhere [28]. For all PCR tests, positive (DNA of pathogen-positive blood samples) and negative (no DNA) controls were included.

Smears of buffy-coat were prepared as described above and stained using May-Grünwald-Giemsa quick stain (Bio-Optica, Milan, Italy). Intracellular inclusions or free amastigote forms of *L. infantum* were searched in each smear by examining the entire stained area at low magnification (×100) and representative areas at high magnification (×1000) for 10 min. All the samples and smears were identified using a unique alphanumerical code, and laboratory personnel conducting the analyses were blind to the treatment groups.

### Entomological survey

**Fig. 2:**
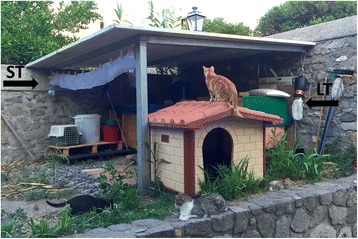
Sand fly trapping in the premises of a household in the island of Lipari. In each of the eight sites, one light trap (*arrow LT*) and sticky traps (*arrow ST*) were monthly set and left working for two consecutive days

Light and sticky traps were used to monitor presence and activity of sand flies during the study period. From May to December 2015, traps were placed monthly in eight different sites (five in Lipari and three in Vulcano). Traps were placed nearby some of the households whose cats were included in the study (Fig. 2). In each site and for each trapping session, one light trap and sticky traps for a total of 2 m^2^ were set and left working for 2 consecutive days (sticky traps), or 2 consecutive nights, i.e. from 6.00 pm to 7.00 am (light traps). Trapping activity was concluded at each site after two consecutive negative trapping sessions. Sand flies collected were separated from other insects with the aid of a stereomicroscope, differentiated by sex and stored into vials containing 70% ethanol according to site and date of capture. Sand fly specimens were prepared for microscopic observation as described elsewhere [29] and identified at species level using morphological keys [30].

### Data management and statistical analyses

A minimum sample size of 80 cats was estimated for each group based on the following assumptions: confidence level: 95%, power: 80%, expected incidence of *L. infantum* infection of 2% and 12% in treated and untreated cats, respectively. In order to make provision for a drop-out of about 20% during the study period, a minimum of 100 cats were included in each group. A cat was considered *L. infantum* infected if it tested positive in at least one of the diagnostic tests employed (IFAT, qPCR on blood and on conjunctival swabs, or buffy coat cytology). The efficacy in preventing *L. infantum* infection was based on the year-crude incidence (YCI), the percentage of infected cats in each group on SD 360 and calculated in each group as follows: YCI = number of infected animals/(number of negative animals included − number of animals not completing the study) × 100.

The difference between YCI in G1 and G2 was tested for statistical significance using Chi-square test. Efficacy in preventing *Leishmania* infection was calculated using the following formula: Efficacy = [(A−B)/A] ×100, where A is % of infected animals in the control group and B is % of animals in the treated group.

Statistical analyses and randomisation were performed using the statistical packages SPSS® 13.0, nQuery + nTerim 3.0 (StatSols), Statistical Solutions® Ltd. 2014, and Microsoft® Excel 2010.

## Results

A total of 204 cats (104 in G1 and 100 in G2), belonging to 80 owners, were enrolled in the study on SD 0. The study population was composed of 111 females (54.4%) and 93 males (45.6%) with age ranging from 6 months to 15 years. During the study 45 cats (25 from G1 and 20 from G2) were removed or lost to follow up for different reasons (e.g. animal lost, collar lost and not replaced within two days, adverse events or suspected adverse drug reaction), whereas 159 animals (79 from G1 and 80 from G2) completed the study (Table 1). Amongst the excluded cats, 18 (8 from G1 and 10 from G2) were removed after the enrolment because found infected by *L. infantum* on samples collected on SD 0. On samples collected at the study closure (SD 360), 5 out of 79 cats in G1, and 20 out of 80 in G2 scored positive to *L. infantum* infection in at least one of the diagnostic tests (Table 2). The majority of animals tested positive by IFAT (15/25; 60%), whereas few cats were positive by qPCR on blood (5/25; 20%), conjunctival swab (1/25; 4%), or both samples (4/25; 16%). Only three cats (3/25; 12%) tested positive to IFAT and qPCR on blood and/or conjunctival swab simultaneously. None of the cats tested positive to cytology on buffy-coat smears either at the inclusion (SD 0) or at the study closure (SD 360). The YCI was 6.3% in G1 and 25.0% in G2 (*χ* ^2^ = 9.095, *df* = 1, *P* = 0.0026) leading to 75% efficacy of the collar in preventing FeL infection. At the study closure all cats were in good general health; however, some of them showed systemic signs such as peripheral lymph node enlargement (G1 = 15.2%; G2 = 35.0%) and splenomegaly (G1 = 5.1%; G2 = 21.3%). Clinical signs were more frequent in animals of the G2 group than in those of the G1 (*χ* ^2^ = 7.266, *df* = 1, *P* = 0.0070).

**Table 1:** Number and characteristics of cats treated with the Seresto® collar (G1) and untreated controls (G2) that either completed or were excluded from the study

	G1	G2	Total
Completed the study			
Number of cats	79	80	159
Number of households	41	39	80
Age (months) Mean (Min - Max)	38.3 (6–180)	29.3 (7–144)	33.7 (6–180)
Gender (%) Female/Male	53.2/46.8	55.0/45.0	54.1/45.9
Weight (kg) Mean (Min - Max)	4.3 (1.9–7.8)	3.9 (2.1–7.0)	4.09 (1.9–7.8)
Hair length (%) Short/Long	81.0/19.0	78.8/21.2	79.9/20.1
Environment (%) Rural/Suburban/Urban	48.1/48.1/3.8	56.2/38.8/5.0	52.2/43.4/4.4
Excluded from the study	G1	G2	Total
Number of cats	25	20	45
Deceased^a^	6	3	9
Suspected adverse drug reaction	1	–	1
Infected by *L. infantum* at the inclusion	8	10	18
Lost to follow up	8	7	15
Collar lost and not replaced promptly	2	–	2

^a^Car trauma (*n* = 4), suspected infectious disease (*n* = 3), respiratory failure (*n* = 1), aortic thromboembolism (*n* = 1)

**Table 2:** Results of serology (IFAT) and qPCR on blood and conjunctival swab for *Leishmania infantum* in cats treated with the Seresto® collar (G1) or in untreated controls (G2) after being exposed to one transmission season in highly endemic area

Group	*n*	IFAT titre		qPCR		Total (%)^a^
		1:80	1:160	Blood	C.S.	
G1	79	2	1	2	1	5 (6.3)^A^
G2	80	9	3	10	5	20 (25.0)^B^

^a^Not the sum per group and row as individual animals tested positive on multiple tests but the total number of individuals testing positive in a group

Significant differences are marked with different upper case letters (*χ* ^2^ = 9.095, *df* = 1, *P* = 0.0026)

*Abbreviation*: *C.S.* conjunctival swab

During the study 18 cats lost the collar once and one twice; collars were replaced within 2 days, except in two cases for which the loss was not reported by the owner, resulting in the exclusion of the animals from the study (Table 1). The collar was well tolerated and few local skin reactions were observed at the application area in four out of the 104 treated cats (3.8%). Of these, one showed mild alopecia, two mild dermatitis and pruritus, and one an ulcerative dermatitis. Except for the latter case for which the collar was removed and the animal excluded from the study and topically treated for (i.e. antibiotic and anti-inflammatory drugs), all the other cases recovered in few days without the need to remove the collar. Heavy flea infestations and the associated itchy dermatitis were recorded in 16 cats of the G2 group; for these animals rescue treatments with a commercial spot-on product containing imidacloprid (Advantage® for cats, Bayer Animal Health GmbH, Monheim, Germany) were authorized on a welfare basis.

Overall, 329 sand flies belonging to three species, namely *P. perniciosus* (*n* = 296; 90.0%), *Phlebotomus neglectus* (*n* = 16; 4.8%), and *Sergentomyia minuta* (*n* = 17; 5.2%) were captured from the end of May to October 2015. The majority of sand flies were captured by light traps (*n* = 297; 90.3%) (Table 3).

**Table 3:** Sites and months of capture of *Phlebotomus perniciosus* in the study area. In each site one light trap and sticky traps for a total of 2 m^2^ were used

Site	Environment	Month of capture					
Lipari 1	Urban		June	July			
Lipari 2	Rural	May		July	August	September	October
Lipari 3	Sub-urban			July	August	September	
Lipari 4	Urban		June				
Lipari 5	Urban					September	
Vulcano 1	Rural	May	June	July	August	September	October
Vulcano 2	Rural	May	June	July	August	September	October
Vulcano 3	Sub-urban				August	September	

## Discussion

The Seresto® collar containing a combination of 10% imidacloprid and 4.5% flumethrin showed to be effective in reducing the risk of infection by *L. infantum* in cats, being thus a tool for controlling FeL in endemic areas. The YCI here recorded in G2, i.e. 25%, was higher than that reported previously in cats (15%) in the same areas [20], but similar to that of dogs (i.e. 27%). Cats included in this trial were at high risk of *L. infantum* infection with the study being carried out in a highly endemic area for FeL. The vast majority of cats lived constantly outdoors in sub-urban or rural areas; in addition, animals of the control group were not treated with any insecticide except in cases of rescue treatments against heavy flea infection. Although cats appear to be more resistant to *L. infantum* than dogs [6], the present data suggest that at least the likelihood of infection in these two hosts is similar as it relies on the risk of being exposed to sand fly bites, also considering that some vectors display a catholic feeding behaviour [17].

Diagnosis of *Leishmania* infection in cats is challenging [14]. The majority of infected cats scored positive to IFAT, but it should be noted that 10 out of 25 infected animals tested positive only to qPCR with blood tissue being more frequently positive (9/10) than conjunctival swab (5/10) (*χ* ^2^ = 2.143, *df* = 1, *P* = 0.1432). Although the comparison of results among different studies is not always possible, our findings are in overall agreement with those reported in previous surveys that combined serological and molecular tests to investigate feline *L. infantum* infection prevalence [20, 31, 32]. On the other hand, conjunctival swabs have recently been considered as a sensitive non-invasive technique for the molecular diagnosis of *L. infantum* infection in both dogs and cats [33–35], displaying positive predictive values in animals with active infection or diseased, and a substantial agreement between serological and molecular tests [34]. In the present study, the purpose of diagnosis was to either discover exposure to infective sand fly bites or active infections for which seroconversion had already occurred. Therefore, the variety of serological and molecular results observed reflects the different infection stages in which exposed animals may be found. According to this variety of patterns, it is strongly advisable to combine serological and molecular diagnostic tests when the purpose of diagnosis is to ascertain exposure to *L. infantum* infection.

In many cases *L. infantum*-infected cats remain apparently healthy, and the progression to clinical illness may be associated with immunosuppressive conditions caused by concurrent diseases. A natural predisposition for a protective cell-mediated immune response pattern to *Leishmania* infection has also been hypothesized for cats [16]. Retroviral infections or other debilitating diseases (e.g. neoplastic diseases) have been sometimes associated with clinical FeL or subclinical *L. infantum* infection [14], but not in a previous study in the Aeolian archipelago where these infections are rare among the examined cat populations [20]. Also, it should be noted that the mean age of enrolled animals was less than three years and the ones testing positive at study closure were most likely infected for the first time. These findings may account for the absence of clinical cases of FeL in positive cats of this study, though leishmaniosis usually evolves as a chronic disease with a long period of incubation [16].

The collar proved to be safe and, with the exception of few local reactions at the collar application site, no adverse events were evaluated as being product related. Local reactions were mainly dermal irritations likely caused by the mechanical rubbing of the collar over the fur and skin of the cats and were similar, for frequency and typology, to those observed in previous studies [22, 23]. All the skin reactions occurred in the first weeks (1–4) after the collar application and healed spontaneously after a slight loosening of the collar with the exception of one case for which the collar was removed to allow a better topical treatment of the lesion. The slow release formulation makes the collar an ideal device for the drug sensitive cat species and allows the use of flumethrin, a potent acaricide with fast acting, repellent properties, due to the differences in metabolic pathway in a species in which it is not possible to apply any of the other current pyrethroids [21]. Additionally, another safe feature of the collar is its safety release system that makes it particularly secure in free roaming cats. Indeed, although all the collared cats enrolled in the study had access to the outdoors, not a single case of hooking or strangling caused by the collar has been observed.

The entomological survey confirmed the presence of competent vectors of *L. infantum* in all the monitored sites, namely *P. perniciosus* and *P. neglectus*, both regarded as the most important vectors of *L. infantum* in the Mediterranean basin. This finding is in agreement with previous surveys conducted recently at the same latitude [29]. Few studies have investigated the sand fly fauna in the Aeolian islands and in the sole survey carried out in the same archipelago (Lipari and Filicudi islands) using sticky traps, *P. perniciosus* was the single species captured [36]. Therefore, the present study complements the number of the sand fly species reported in the archipelago with other two species, one of which (*P. neglectus*) is a proven vector of *L. infantum*. Interestingly, during the survey *P. perniciosus* was found in sites featured by different environments, i.e. urban, peri-urban and rural. The longest presence and activity of *P. perniciosus* was however recorded in rural sites of both Lipari and Vulcano islands with a constant activity from late May to October and peaks in July and August. This may represent the period of higher risk of exposure to *L. infantum* infection, especially in the middle of summer, when tourists and their animals come in large numbers to spend holidays in these islands.

## Conclusions

This study shows that the Seresto® collar containing a combination of imidacloprid and flumethrin is safe and effective in reducing the risk for feline *L. infantum* infection. This collar currently represents the only possible preventive measure for FeL. Treatment should strategically be adopted either for providing individual protection to cats living in or travelling to *L. infantum* endemic areas, or for reducing the potential of infected cats to act as reservoirs of the pathogen.

## Abbreviations

DNA: Deoxyribonucleic acid
EDTA: Ethylenediaminetetraacetic acid
FeL: Feline leishmaniosis
FeLV: Feline leukemia virus
FIV: Feline immunodeficiency virus
IFAT: Immunofluorescence antibody test
qPCR: Quantitative real-time polymerase chain reaction
SD: Study day
VBDs: Vector-borne diseases
YCI: Year crude incidence
